# Abnormal Expression of Dysferlin in Blood Monocytes Supports Primary Dysferlinopathy in Patients Confirmed by Genetic Analyses

**DOI:** 10.3389/fneur.2020.540098

**Published:** 2021-02-04

**Authors:** Huili Zhang, Yaqin Li, Qiusheng Cheng, Xi Chen, Qiuxia Yu, Ze Li

**Affiliations:** ^1^Department of Neurology, Guangzhou First People's Hospital, School of Medicine, South China University of Technology, Guangzhou, China; ^2^Department of Neurology, The Seventh Affiliated Hospital, Sun Yat-sen University, Shenzhen, China; ^3^Prenatal Diagnostic Center, Guangzhou Women and Children's Medical Center, Guangzhou Medical University, Guangzhou, China

**Keywords:** dysferlinopathy, *DYSF*, MRI, dysferlin, peripheral blood mononuclear cells

## Abstract

**Objective:** Dysferlin deficiency causes dysferlinopathy. This study aimed to expand the mutational spectrum of dysferlinopathies, to further study one case with diagnostic ambiguity, and to identify the diagnostic value of dysferlin expression in total peripheral blood mononuclear cells (PBMC).

**Methods:** The clinical and molecular profiles of dysferlinopathies in eight Chinese patients were evaluated. We also conducted magnetic resonance imaging (6/8) and determined dysferlin protein expression in muscle (7/8) and PBMC (3/8).

**Results:** Nine of the 13 *DYSF* mutations identified were novel. One patient was homozygous for the Gln111Ter mutation by genomic DNA sequencing but was found to be heterozygous by sequencing of cDNA from total PBMC. A daughter of this patient did not carry any Gln111Ter mutation. Abnormal muscle MRI with predominant involvement of the medial gastrocnemius and soleus muscle was observed in 5/6 patients. Dysferlin levels were significantly reduced (immunohistochemistry/immunoblot) or absent (immunohistochemistry) in muscle and total PBMC (26–39%) for most patients. Sarcoplasmic accumulation of dysferlin was detected in one patient.

**Conclusion:** Genomic DNA sequencing detects frequent homozygous mutations, while fewer heterozygous mutations in cDNA are detected after posttranscription. Total PBMC may serve as an alternative to confirm diagnosis and to guide further testing in dysferlinopathies. Our results contribute to the mutational spectrum of dysferlinopathies.

## Introduction

Dysferlinopathies are autosomal recessive muscular dystrophies caused by mutations in *DYSF* (1). Three classic phenotypes have been described including Miyoshi myopathy (MM; OMIM no. 254130), limb girdle muscular dystrophy 2B (LGMD2B; OMIM no. 253601), and distal anterior compartment myopathy. In addition, other intermediate proximodistal phenotypes, pseudometabolic myopathy, and hyperCKemia also occur (2, 3).

*DYSF*, a large gene (>230 kb) located on chromosome 2p13, contains 55 exons (1). To date, over 400 disease-causing mutations have been identified and logged in the UMD-DYSF website **(**www.umd.be/DYSF/) (4). Furthermore, deep intronic mutations can also be a common underlying cause of dysferlinopathy (5). Because these mutations are not concentrated at any “mutational hot spot” (2, 3), *DYSF* mutation analysis remains challenging and time-consuming.

Dysferlin is encoded by *DYSF* and is expressed predominantly in the sarcolemma of the skeletal muscles. In addition to skeletal muscle, dysferlin is expressed in other tissues, and the dysferlin expression level in blood monocytes can be used as a diagnostic tool for dysferlinopathy (6). Although reliable, quantification in total peripheral blood mononuclear cells (PBMC) remains controversial (7), and the use of total PBMC is inexpensive, quick, and less invasive for clinical diagnosis.

As mentioned above, mutations in *DYSF* cause a spectrum of phenotypes, but the factors responsible for these distinct pathologies are unknown. One approach to identify the underlying mechanisms is to study patient cohorts from different ethnic origins. Different *DYSF* mutations have been identified in various ethnicities (8–10), although few studies focused on Chinese patients (11, 12).

The aim of this study was to define the *DYSF* mutational and phenotypic spectra in Chinese dysferlinopathies. We describe the clinical, imaging, pathological, and molecular data of eight dysferlinopathy patients from seven unrelated Chinese families. We also emphasize the cautious use of total PBMC as an alternative to muscle biopsy owing to quicker turnover and non-invasiveness.

## Materials and Methods

### Patients

This study included four men and women with dysferlinopathy. All eight patients came from Guangzhou First People's Hospital. All eight patients were of Chinese origin and from seven different families without any known consanguinity or familial history of neuromuscular disease. The clinical and laboratory findings, including routine blood tests, electromyography (EMG), and routine cardiological assessments of each patient were retrospectively reviewed. Besides, magnetic resonance imaging (MRI) of muscle, immunohistochemistry (IHC) of muscle, immunoblotting of dysferlin expression in total PBMC, and/or muscle and genetic analysis were obtained from some patients after informed consent. The control muscle samples were taken from patients with informed consent who had undergone open fracture surgery as a result of a car accident. The control PBMC sample was taken from patient 1's sister with informed consent since she is a non-carrier of *DYSF* mutations.

### MRI

MRI was performed using a 1.5-T MRI system (Gyroscan Intera; Philips, Best, The Netherlands) with a body coil. The imaging protocol included a conventional T1-weighted spin echo sequence (TR = 500; TE = 18) and a STIR sequence (TR = 3,462; TE = 70; TI = 150) in 10-mm slices. STIR sequences were used to detect myoedema.

### IHC

Frozen skeletal muscle biopsy sections of the gastrocnemius or quadriceps muscle were processed for histochemistry and IHC using standard methods. Monoclonal antibodies specific to dysferlin, dystrophin (DYS1, DYS2, and DYS3), and sarcoglycan (α, β, γ, and δ), all from Novocastra (Newcastle upon Tyne, UK), were used.

### Immunoblotting

Dysferlin expression in total PBMC from patients and relatives where available were evaluated as previously described (7). Briefly, 50 μg of the samples was loaded onto the gel, and the resulting blot was incubated with a rabbit monoclonal antibody to dysferlin (Abcam, Cambridge, MA) (1:5,000 dilution) and a mouse monoclonal antibody to GAPDH (Abcam, Cambridge, MA) (1:5,000 dilution) as a loading control. The relative dysferlin expression (the ratio = dysferlin expression/GAPDH expression) was determined for each sample. Then, we assigned a value of 100% to a healthy individual with no mutations in *DYSF* as a positive control. The relative ratio was determined in other samples when compared with that of the control and expressed as a percentage.

Dysferlin expression in muscle from patients and healthy individuals was also evaluated by immunoblotting using a rabbit monoclonal antibody to dysferlin (1:8,000; Abcam, Cambridge, MA) using the standard method. GAPDH (1:5,000; Abcam, Cambridge, MA) was used as a loading control.

### Genetic Analysis

Genomic DNA was extracted from the peripheral blood by using a DNA extraction kit per the manufacturer's instructions (Qiagen, Hilden, Germany). The 55 exons and adjacent introns of *DYSF* were PCR-amplified. The PCR products were sequenced directly on an ABI Prism 3100 Genetic Analyzer (Applied Biosystems, Foster City, CA). All sequences were aligned to the *DYSF* genomic sequences in the GenBank database (Gene ID: NM_003494.2) using the Sequencer alignment software (Gene Codes Corp, Ann Arbor, USA). Patients' relatives were tested for specific *DYSF* mutations detected in the propositus of each family. Additionally, 200 control chromosomes from unaffected individuals of the same ethnic background were tested for the specific *DYSF* mutations detected in the propositus.

Mutation analyses of dysferlin cDNA from total PBMC of patient 1's family and that from muscle tissue of patient 1 were performed as described above.

### *In silico* Analysis

The identified sequence mutations were compared to those in the dbSNP database (http://www.ncbi.nlm.nih.gov/SNP), Human Genome Mutation Database (http://www.hgmd.cf.ac.uk/ac/), Leiden dysferlin-specific database (www.lovd.nl/DYSF/), and UMD-DYSF database (www.umd.be/DYSF/) to determine whether they had been previously reported as benign or disease-causing mutations.

Bioinformatics analyses using SIFT (http://sift.jcvi.org/), PANTHER (http://www.pantherdb.org/), MutPred (http://mutpred.mutdb.org/), and UMD-Predictor (http://www.umd.be) were used to predict the effects of the DNA mutations on protein structure and function.

### Paternity Test

After informed consent was obtained, peripheral blood extracted from the father, mother, and sister of patient 1 was sent to Sun Yat-sen University's forensic center for paternity testing.

## Results

### Mutational Analysis of the DYSF Gene

Genetic analysis of the *DYSF* coding sequence in eight patients from seven unrelated families identified 13 mutations, of which 69% (9/13) were novel. These mutations were scattered across the gene without any mutational “hot spots” and included three frameshift, five non-sense, and five missense mutations. Three patients carried a single homozygous mutation even though they were not born to consanguineous parents, and five patients had a compound heterozygous mutation. All these mutations are presented in Table 1. One mutation occurred as homozygous and heterozygous changes in patient 1-F and patient 1, respectively, and is discussed below.

**Table 1:** Summary of mutations identified in eight patients with dysferlinopathy.

**Patient no**.	**Position (GRch37/hg19)**	**Exon**	**Nucleotide change**	**Protein change**	**Mutational event**	**State**
1	chr2:71730438	4	c.331C>T	p.Gln111Ter	Non-sense	Heterozygous
	chr2:71909744	54	**c.6141delC**	stop at 2073 (exon 55)	Frameshift	Heterozygous
1-F	chr2:71730438	4	c.331C>T	p.Gln111Ter	Non-sense	Homozygous
2	chr2:71789030	23	**c.2311C>T**	p.Gln771Ter	Non-sense	Heterozygous
	chr2:71797009–71797013	27	**c.2870-2874delAGACC**	stop at 968	Frameshift	Heterozygous
3	chr2:71778765	19	**c.1667T>C**	p.Leu556Pro	Missense	Heterozygous
	chr2:71838459	37	c.3988C>T	p.Gln1330Ter	Non-sense	Heterozygous
	chr2:71887771	44	**c.4876G>A**	p.Val1626Ile	Missense	Heterozygous
4	chr2:71795311	26	**c.2653G>T**	p.Glu885Ter	Non-sense	Homozygous
5	chr2:71778203	18	c.1555G>A	p.Gly519Arg	Missense	Heterozygous
	chr2:71895900	48	**c.5357G>A**	p.Trp1786Ter	Non-sense	Heterozygous
6	chr2:71766328–71766331	16	**c.1439–1442delTGAG**	stop at 491	Frameshift	Homozygous
7	chr2:71753461	12	**c.1165G>A**	p.Glu389Lys	Missense	Heterozygous
	chr2:71838474	37	c.4003G>A	p.Glu1335Lys	Missense	Heterozygous

*The novel mutations are indicated in bold*.

The bioinformatics analyses predicted the effect of the mutations on protein structure and function. Three frameshift and five non-sense mutations were certainly disease causing because they translated into a truncated protein. Of the five missense mutations, three showed consistent results. The remaining two missense mutations were predicted to be pathogenic by the SIFT and MutPred programs but appeared as polymorphisms when using the PANTHER and UMD-Predictor programs. The bioinformatics data are shown in Table 2.

**Table 2:** *In silico* prediction of mutation effect on protein function^a^.

**Mutation**	**SIFT**	**Panther^**b**^**	**UMD-predictor**	**MutPred** ^**c**^	
				**Deleterious mutation**	**Top 1 of molecular**
				**probability**	**mechanism disrupted**
c.1667T>C^d^	Damaging	p deleterious = 59.12%	Probably pathogenic	*g* = 0.661	Loss of stability (*P* = 0.0036)^*^
c.4876G>A^d^	Tolerated	p deleterious = 20.48%	Polymorphism	*g* = 0.495	Gain of sheet (*P* = 0.0827)
c.1555G>A	Damaging	p deleterious = 91.05%	Pathogenic	*g* = 0.561	Loss of sheet (*P* = 0.0817)
c.1165G>A^d^	Damaging	p deleterious = 39.82%	Polymorphism	*g* = 0.632	Gain of MoRF binding (*P* = 0.0387)^*^
c.4003G>A	Damaging	p deleterious = 46.69%	Probable polymorphism	*g* = 0.845	Gain of MoRF binding (*P* = 0.0078)^*^

a *Four bioinformatics tools, namely, SIFT, PANTHER, MutPred, and UMD-Predictor, were used*.

b *p deleterious > 50% is considered probably pathogenic*.

c *g > 0.5 and p < 0.05 are considered probably pathogenic; g > 0.75 and p < 0.05 are considered pathogenic; g > 0.75 and p < 0.01 are considered certainly pathogenic;*

* *p < 0.05*.

d *Novel mutation*.

### DYSF Gene Analysis in Patient 1's Family

**Figure 1:**
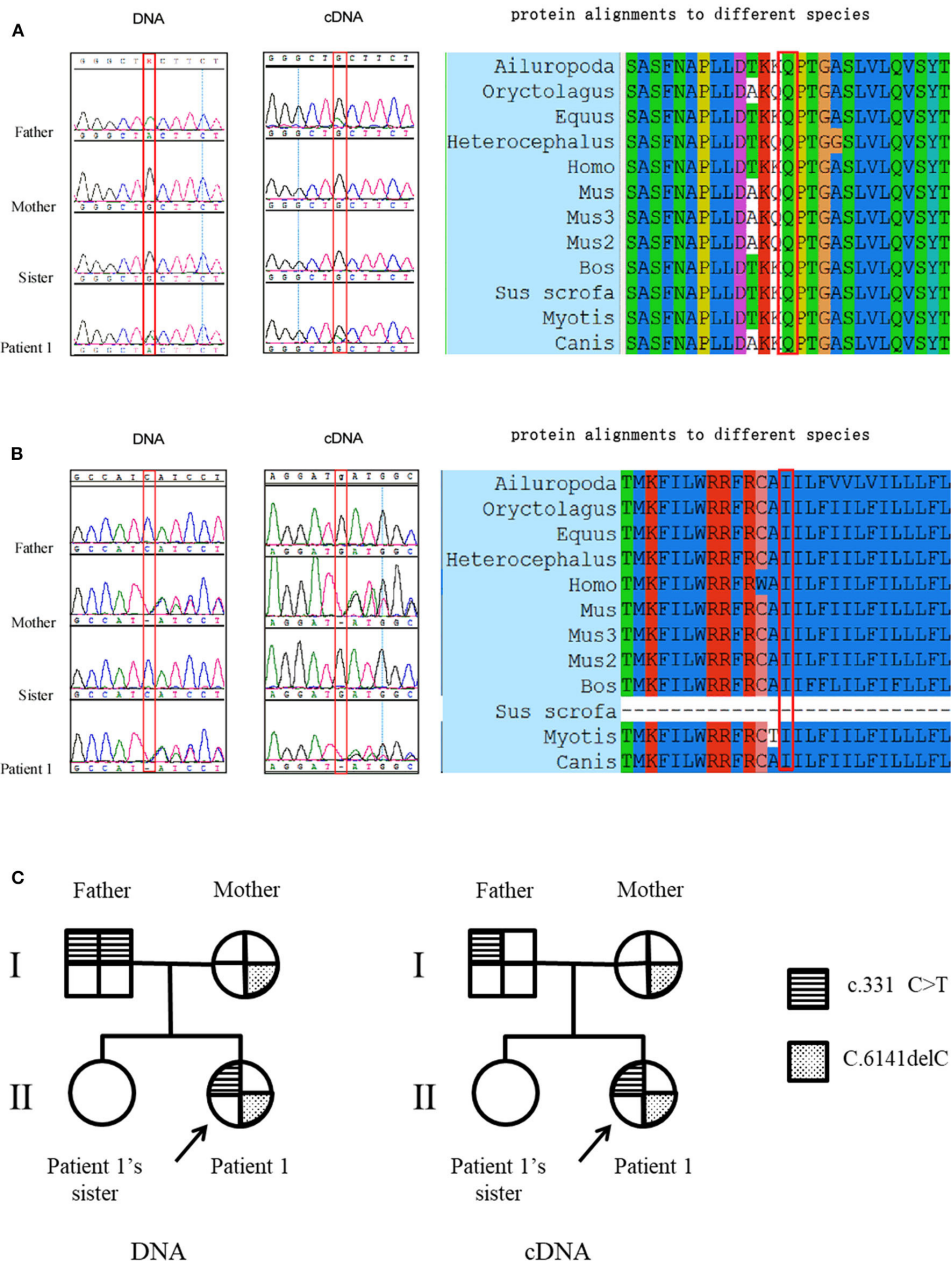
Mutation studies of patient 1's family. **(A)** c.331C>T (p.Gln111Ter) mutation analysis in genomic DNA and total PBMC cDNA, together with protein alignments to different species. Note that the father was found to be homozygous for the c.331C>T mutation in genomic DNA while he was found to be heterozygous for the c.331C>T mutation from the total PBMC cDNA. **(B)** c.6141delC (p.Cys601Stop) mutation analysis in genomic DNA and total PBMC cDNA, together with protein alignments to different species. The protein alignments indicate that these two mutations occur in highly conserved amino acids. **(C)** The pedigree of patient 1's family with representation of the segregation of mutations.

Patient 1 carried a compound heterozygous mutation: c.331C>T and c.6141delC (Figures 1A,B). The patient's father (patient 1-F) carried a single homozygous c.331C>T mutation, and the mother was heterozygous for the c.6141delC mutation. The patient's sister did not carry any allele for the c.331C>T or c.6141delC mutation (Figures 1A,B). Gene analysis was repeated after 6 months with new peripheral blood samples from the family to validate the results described above (data not shown). The paternity test confirmed that the patient's sister was the biological offspring of the parents, thus eliminating any doubt regarding the findings (data not shown). Genetic testing using only dysferlin cDNA from total PBMC authenticated all the mutations found in the genomic analysis except for the c.331C>T mutation detected in the father (patient 1-F). From the total PBMC cDNA, the father was found to be heterozygous for the c.331C>T mutation, rather than having the homozygous mutation observed by the genomic DNA mutation study (Figure 1C). Furthermore, genetic testing using muscle dysferlin cDNA from patient 1 confirmed that she carried a compound heterozygous mutation: c.331C>T and c.6141delC. The c.6141delC mutation occurs in exon 54, causing a frameshift (stop at 2,073 bp) that leads to termination of translation and consequently a truncated protein (data not shown).

### Clinical Analysis

All eight patients showed significant elevation of creatine kinase (CK) levels in this study. Except for patient 1-F, all patients showed a myopathic pattern in needle EMG (data not shown). Cardiac evaluations including serum brain natriuretic peptide (BNP) determination, electrocardiograms (ECGs), and echocardiography showed that only patient 4, with disease duration of 4 years, had sinus tachycardia and a short PR period in ECG. Patient 4 also demonstrated anterior mitral valve prolapse (MVP) and slight mitral insufficiency in ultrasonic cardiography (UCG) (data not shown). A detailed clinical report of the patients is presented in Table 3.

**Table 3:** Detailed clinical description of patients with dysferlinopathy.

**Patient no**.	**Sex/age, year**	**Onset, year**	**Duration, year**	**Phenotype**	**First symptoms or signs^**a**^**	**Muscle weakness**				**Muscle atrophy^**b**^**	**Accompanying symptoms or signs**
						**PLL**	**PUL**	**DLL**	**DUL**		
1	F/18	14	4	MM	I	Y+	N	Y++	N	DLL(S)	Symmetric hypertrophy of the quadriceps
1-F	M/52	NA	NA	Isolated hyperCKaemia	NA	N	N	N	N	NA	NA
2	M/39	19	20	DACM	C/H	Y++	Y+	Y+++	N	PLL, DLL(S)	Ankle contracture
3	M/20	17	3	Proximodistal	I	Y+	N	Y++	N	DLL(S)	Flatfoot
4	F/33	30	3	Proximodistal	U	Y++	N	Y+++	N	DLL(S)	N
5	F/22	20	2	LGMD2B	M/U	Y+	N	N	N	N	Congenital amblyopia, hexadactyly
6	M/18	16	2	MM	T	Y+	N	Y++	N	DLL(S)	N
7	F/42	41	1	MM	T	N	N	Y++	N	N	N

*MM, Miyoshi myopathy; DACM, distal anterior compartment myopathy; LGMD2B, limb girdle muscular dystrophy 2B; PLL, proximal lower limb; PUL, proximal upper limb; DLL, distal lower limb; DUL, distal upper limb; Y, observed; N, not observed; +, mild; ++, moderate; +++, severe; NA, not available*.

a *I, exercise intolerance; C, calf atrophy in the absence of weakness; H, difficulties standing on heels; U, difficulties walking uphill or upstairs; M, exercise-induced myalgia; T, difficulties standing on toes*.

b *(S), symmetric*.

### Muscle MRI Detection of Patients With Dysferlinopathy

**Figure 2:**
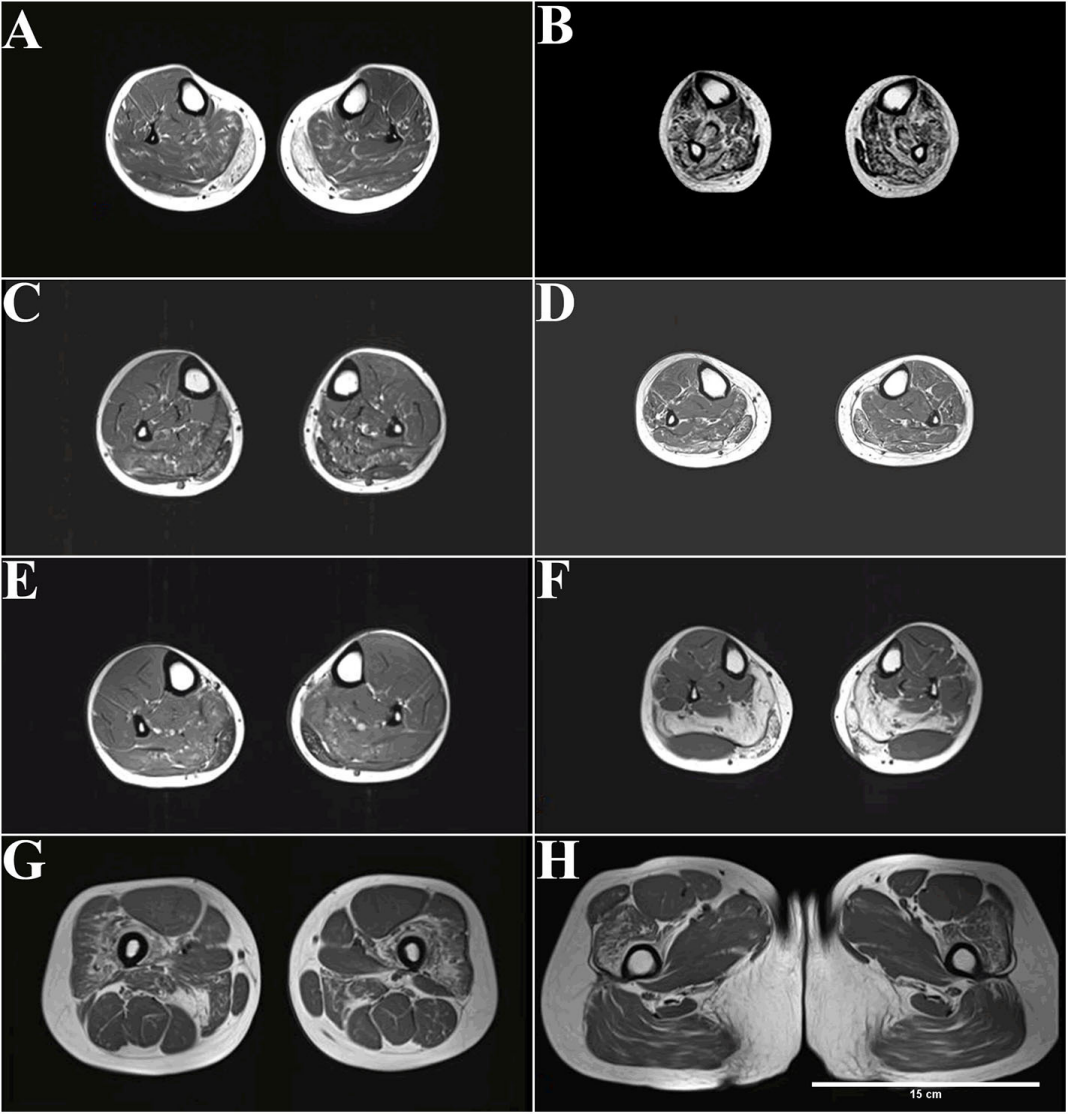
MRI (T1-weighted) of patients with dysferlinopathy shows fatty infiltration of muscle. **(A)** The peroneus, extensor digitorum longus, and extensor halluces longus muscles were involved in patient 1 with disease duration of 4 years. **(B)** More advanced stages show an involvement of the tibialis anterior in patient 2 with disease duration of 20 years. **(C,D)** The peroneus, extensor digitorum longus, and extensor halluces longus muscles were also involved in patient 3 **(C)** and patient 4 **(D)** with disease duration of 3 years. **(E,F)** Only the gastrocnemius and soleus muscles were affected in patient 6 **(E)** and patient 7 **(F)** with disease duration of 1–2 years. **(G,H)** Increased signal can be observed in the quadriceps femoris and adductor magnus muscle in patient 7.

Axial T1-weighted MRI showed fatty infiltration and/or atrophy in the lower extremities of all patients except for patient 1-F and patient 5 (Figure 2). STIR sequences did not detect any myoedema (data not shown).

The peroneus, extensor digitorum longus, and extensor halluces longus muscles were involved in patient 1 with disease duration of 4 years (Figure 2A). More advanced stages show an involvement of tibialis anterior in patient 2 with disease duration of 20 years (Figure 2B). The peroneus, extensor digitorum longus, and extensor halluces longus muscles were also involved in patient 3 (Figure 2C) and patient 4 (Figure 2D) with disease duration of 3 years. Only the gastrocnemius and soleus muscles were affected in patient 6 (Figure 2E) and patient 7 (Figure 2F) with disease duration of 1–2 years. Increased signal can be observed in the quadriceps femoris and adductor magnus muscle in patient 7 (Figures 2G,H).

Distal anterior compartment myopathy (DACM) is a rare dysferlinopathy phenotype with distinguishable MRI features; the anterior rather than the posterior muscles of the lower legs are predominantly affected. Despite patient 2's diagnosis of DACM, nearly all lower leg muscles were involved because of the long course of the disease (Figure 2B). Interestingly, completely fatty infiltration was observed in the medial gastrocnemius muscle, whereas a normal signal was observed in the lateral gastrocnemius muscle in patient 7, who had late-onset disease with a 1-year duration (Figure 2F).

Despite the short disease duration, patients with late onset, i.e., after 30 years old, displayed severe degeneration of the lower legs on MRI. For example, patient 4, with 3-year disease duration, displayed more widespread distribution muscles with fatty infiltration (Figure 2D). In addition, patient 7, with 1-year disease duration, showed grade 4 fatty infiltrations in the medial gastrocnemius muscles and soleus muscle (Figure 2F).

MRI shows impaired proximal and distal muscles at onset in MM. For example, patient 7 was initially diagnosed with MM, but fatty infiltration of the proximal thigh muscles (adductor magnus muscle and quadriceps femoris) was also observed (Figures 2G,H). MRI can be used to detect muscle alteration, years before disease onset. For example, striking signal alterations were observed in the proximal thigh muscles (adductor magnus muscle and quadriceps femoris) before patient 7 had trouble climbing stairs (Figures 2G,H).

### Dysferlin Expression in Patient Skeletal Muscles

**Figure 3:**
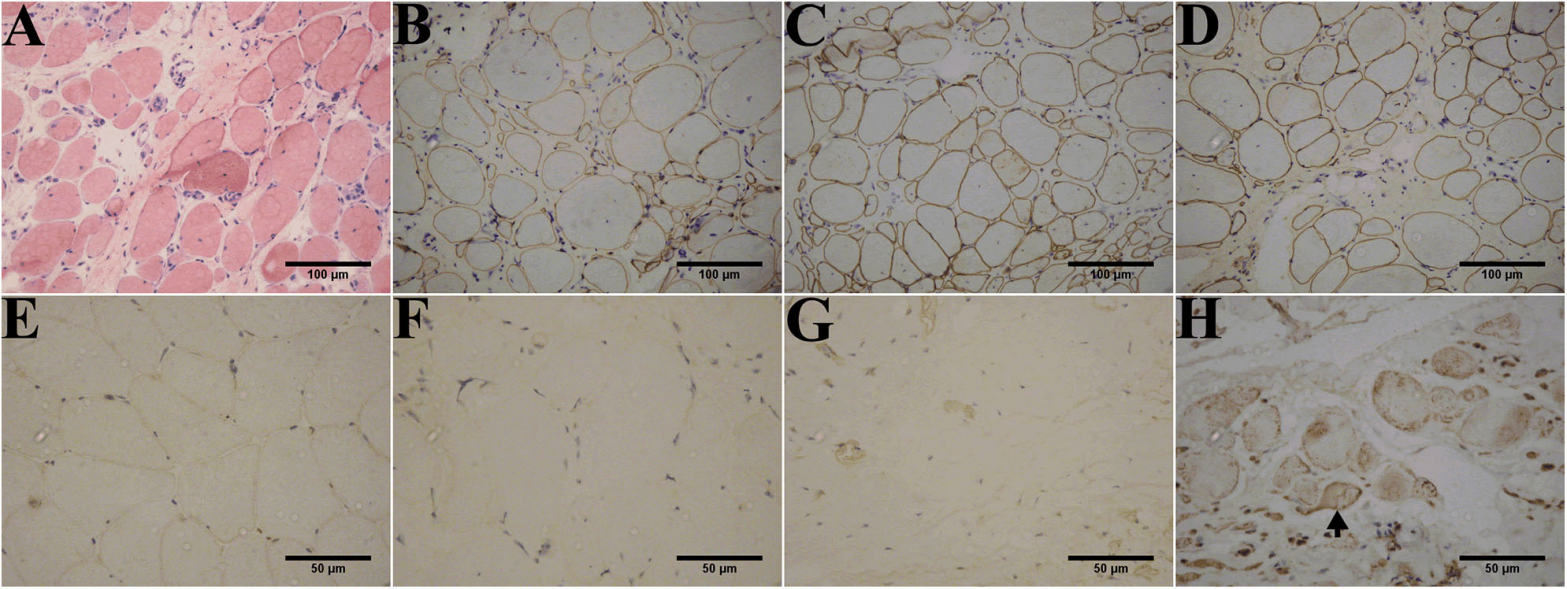
Immunohistochemistry of dysferlinopathy patients. **(A)** H&E staining of muscle tissue from patient 1 showed myopathic or dystrophic alterations. Notice the increase of internal nuclei, variation in fiber size, endomysial mononuclear infiltration, and increase in inter- and intra-fascicular connective tissues. **(B–E)** Immunohistochemistry showed normal staining for DYS1 **(B)**, DYS2 **(C)**, and DYS 3 **(D)** in patient 1 and normal dysferlin staining **(E)** in a healthy individual. Immunohistochemistry indicated deficiency **(F)** or absence **(G)** of dysferlin expression in the sarcolemma and sarcoplasm from patients 1 and 2. **(H)** Aberrant aggregation of dysferlin in the sarcoplasm was detected in patient 3. Arrows point to fibers with positive dysferlin staining in the sarcolemma along with cytoplasmic dysferlin accumulation. The control muscle samples were taken from healthy individuals with informed consent who had undergone open fracture surgery as a result of a car accident.

Muscle biopsies obtained from all patients except patient 1-F displayed three patterns of dysferlin immunoreactivity: [1] general reduction of sarcolemmal expression (Figure 3F), [2] total absence of dysferlin expression at the sarcolemma and sarcoplasm (Figure 3G), and [3] sarcoplasmic granular accumulation (Figure 3H). Dysferlin staining can occur at the sarcolemma when accompanied by sarcoplasm accumulation in scattered fibers (Figure 3H). In addition, normal dystrophin (Figures 3B–D), dysferlin (Figure 3E), and sarcoglycan (data not shown) expressions were observed.

**Figure 4:**
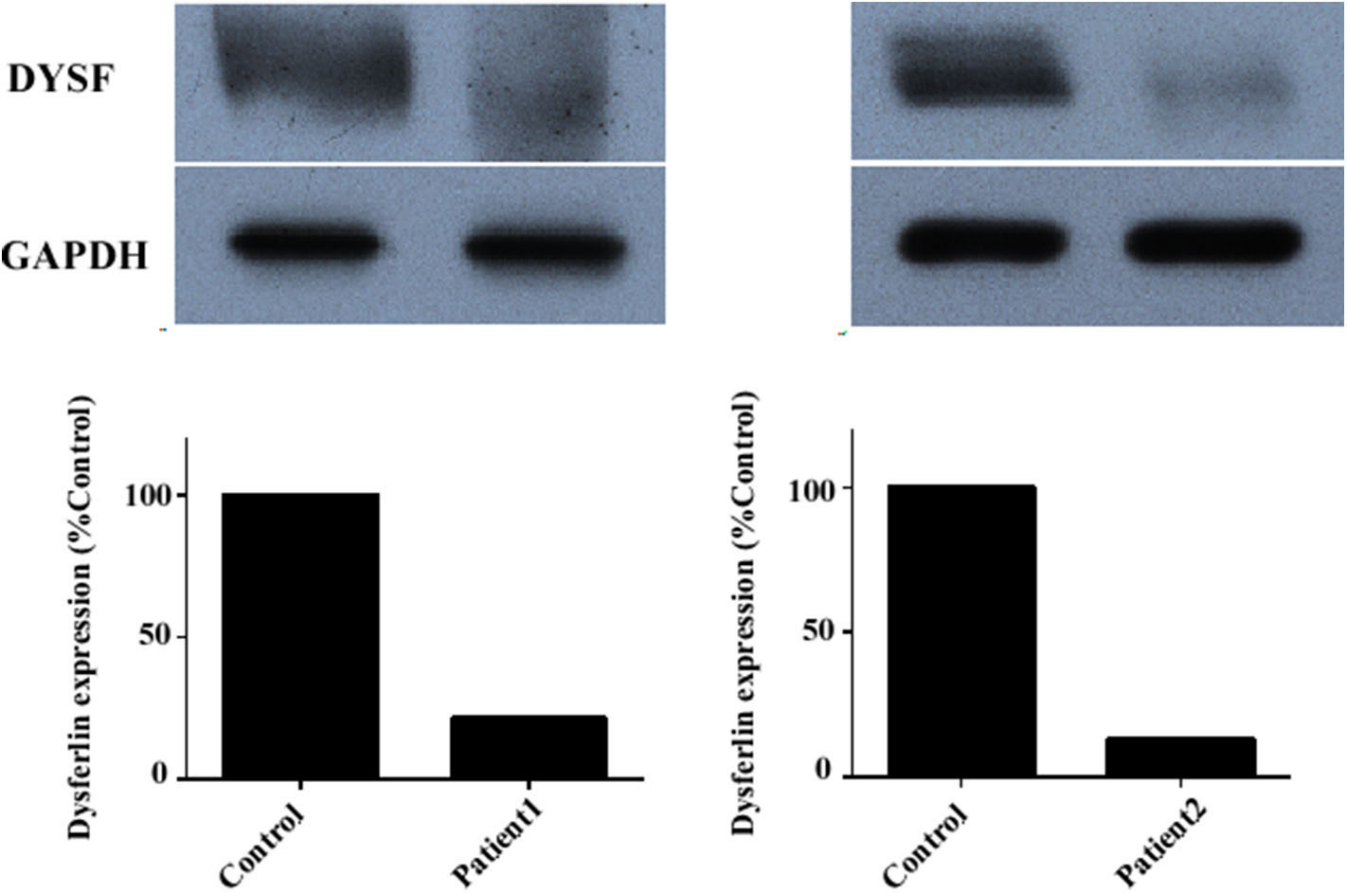
Dysferlin expression in muscle samples of two representative dysferlinopathy patients. Dysferlin expression (upper panel) was significantly reduced in patient 1 and patient 2 compared with healthy individuals. The lower panel shows GAPDH loading control. The data are also graphically presented. The control muscle samples were taken from healthy individuals with informed consent who had undergone open fracture surgery as a result of a car accident.

Immunoblotting of muscle tissue showed a reduction in dysferlin expression in patients (Figure 4). The relative dysferlin levels in patients were 13–22% of those in healthy individuals. The control muscles of healthy individuals were from normal muscle sample bank in our clinical center. Neither the type of mutation nor the phenotype correlated with the amount of detectable dysferlin.

### Dysferlin Expression in Total PBMC

**Figure 5:**
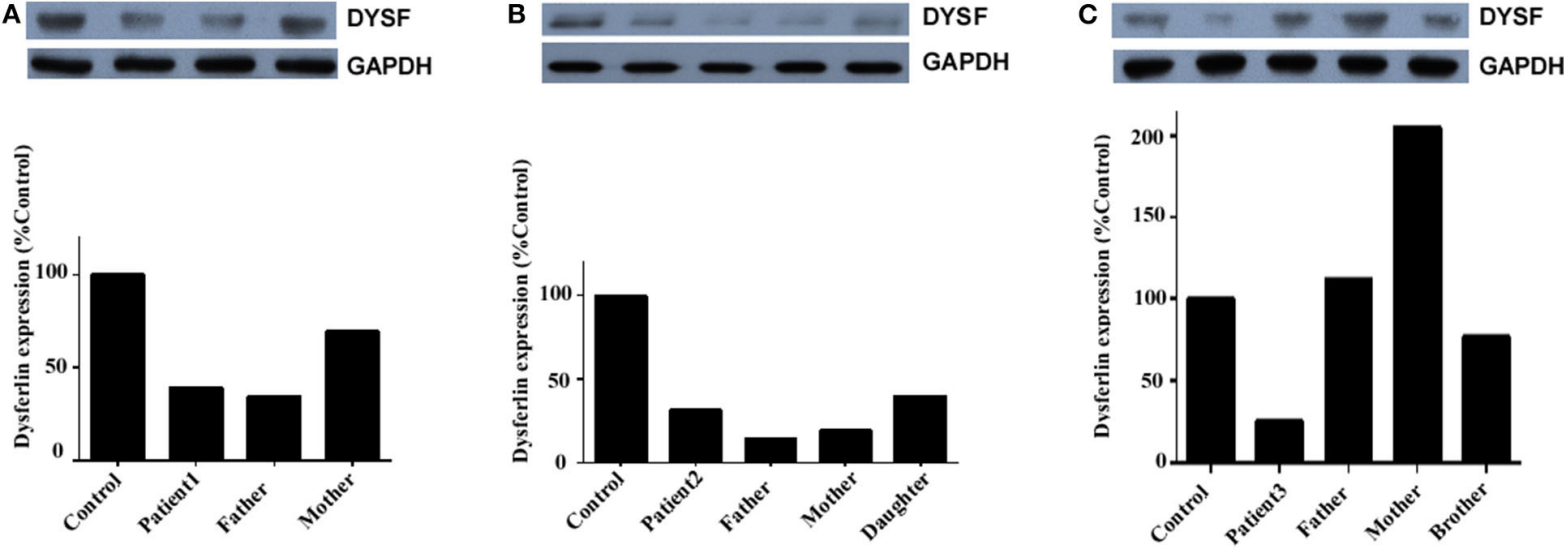
Dysferlin expression in total PBMC of three unrelated families. **(A)** Relative dysferlin expression (upper panel) in patient 1's family. The father was also a patient (patient 1-F), and the mother was a carrier. **(B)** Relative dysferlin expression (upper panel) in patient 2's family. The father, mother, and daughter were carriers. **(C)** Relative dysferlin expression (upper panel) in patient 3's family. The father, mother, and brother were carriers. GAPDH (lower panel) was used as a loading control. The data are also graphically presented. The control PBMC sample was taken from patient 1's sister with informed consent since she is a non-carrier of *DYSF* mutations.

Dysferlin expression was determined in the total PBMC of three unrelated families: those of patient 1, patient 2, and patient 3 (Figure 5). In addition to the propositus, blood was collected from the parents, children, or siblings of the patients, and mutation analysis was used to evaluate the carrier status (data not shown). Because patient 1's sister was the only healthy person with no *DYSF* mutations, her blood sample served as control for all three families.

Patient 1, her father, and her mother (carriers) had relative dysferlin expressions of 39, 34, and 70%, respectively (Figure 5A), whereas those in patient 2 and patient 2's father, mother, and sibling (carriers) were 32, 14, 19, and 40%, respectively (Figure 5B), and those in patient 3 and patient 3's father, mother, and sibling were 26, 112, 205, and 77%, respectively (Figure 5C). Of note, two individuals (patient 2's father and mother) who had a dysferlin level <20% were asymptomatic heterozygotes. From the same patient, dysferlin expression in muscle seems to be lower than that in the total PBMC. However, only three patients determined the dysferlin expression in total PBMC, we did not perform further statistical analysis. The sample size of total PBMC detection from patients will need to be expanded in the future to study a correlation between immunoblotting studies on muscle and blood.

## Discussion

In this study, we identified 13 dysferlin gene mutations, of which nine are novel. Although some studies focused on Chinese patients (11, 12), there is no founder mutation reported in this population. The new frameshift and non-sense mutations will cause dysferlinopathy because of the predicted truncation of the protein that will likely result in a complete loss of protein function. Within the group of patients with missense mutations, these mutations, excluding the c.4876G>A, p.Val1626Ile mutation in patient 3, will likely have deleterious effects. First, these mutations are not polymorphisms because each mutation was present in only one patient and not found in the 200 chromosomes from normal individuals. Second, these mutations are located in a highly conserved domain within the ferlin family of proteins (13) (data not shown). Third, these mutations are pathogenic based on bioinformatics analyses. Finally, two of these mutations have been reported as being causal for the disease (14).

In contrast, the c.4876G>A, p.Val1626Ile mutation detected in patient 3 is likely to be non-pathogenic. First, it was present not only in patient 3 but also in the healthy relatives (the father and brother); thus, it may be a polymorphism. Second, the father and brother were found to carry one allele for the c.3988C>T mutation (non-sense mutation) and another allele for the c.4876G>A mutation (missense mutation). If the latter mutation was pathogenic, both the father and brother would have the disease because of the compound heterozygous nature of the mutation. However, the father and brother did not display any weakness or muscle atrophy and had normal CK levels (data not shown). Thus, the pathogenicity of the c.4876G>A mutation can be excluded. Based on the bioinformatics analyses, we propose that compound heterozygous mutations in exon 39 (c.3988C>T, p.Gln1330Ter) and exon 21 (c.1667T>C, p.Leu556Pro) caused the disease in patient 3's family.

Similar to other studies, a correlation between genotype and phenotype was not observed in this study (15). For instance, patients with non-sense mutations displayed a heterogeneous phenotype (MM, LGMD2B, DACM, or isolated hyperCKaemia). Moreover, patients with missense mutations did not present with more severe disease than those with truncating mutations (2). Finally, truncating mutations or absence of dysferlin did not trigger an earlier disease onset compared to other missense mutations or partial deficiency (14).

Patient 1-F was homozygous for the c.331C>T (p.Gln111Ter) mutation. The C-T transition changes glutamic acid into a stop codon, leading to premature termination of translation at codon 111. This mutation was observed in a heterozygous state in a French woman and reported as pathogenic (16). However, in the present study, patient 1-F showed no weakness or atrophy except for a high CK value. The relative dysferlin expression in total PBMC was significantly decreased (39% of that in the normal control). Thus, we speculate that patient 1-F has as a rare phenotype called isolated hyperCKaemia in dysferlinopathy. It is possible that an abnormality could be detected in muscle MRI and/or biopsy, but in the case of patient 1-F, these procedures were denied.

Although patient 1-F was homozygous for the c.331C>T (p.Gln111Ter) mutation, one of his daughters (patient 1's sister) did not carry any allelic mutation for the c.331C>T mutation. Furthermore, patient 1-F was found to be homozygous for the c.331C>T mutation by genomic DNA analysis but heterozygous when dysferlin cDNA from total PBMC was analyzed. Several reasons may explain for this discrepancy. First, although patient 1's sister inherited one allele for the c.331C>T mutation from her father, spontaneous mutation of c.331 T>C occurred. Second, the dysferlin cDNA in the total PBMC was different from that in muscle, as 14 unique *DYSF* transcript variants involving exons 5a, 17, and 40a exist (17). It is possible that one predominant transcript variant exists in total PBMC, whereas another exists in muscle. Finally, the discrepancy is unexplained and might have been addressed by testing other tissues in the father, but unfortunately, the father declined to provide additional samples. Therefore, interpretation of cDNA mutational analysis of dysferlinopathy should be cautious.

We reiterate that genotype is not a predictor of phenotype or disease severity. However, some manifestations must be addressed:

(1) MM onset is between 12 and 30 years, but patient 7 had late onset, shortly after pregnancy at 41 years, with acute initial weakness and severe progression. Post-pregnancy hormonal fluctuations may account for the disease severity (15).

(2) Similar to a Japanese case (18), the DACM patient had early ankle contractures, although it needs to be established whether this is a unique DCAM pathology.

(3) The cardiac involvement in dysferlinopathies needs to be monitored during the course of the disease (19). In this study, one subject (patient 4) had milder cardiac involvement.

(4) Misdiagnosis of polymyositis is an obstacle in LGMD2B therapy (20). In this study, two patients misdiagnosed with polymyositis received corticosteroids for years. Therefore, the clinicians must be vigilant regarding steroid-resistant polymyositis.

Dysferlinopathy is a highly heterogeneous disorder. Consequently, expression of muscle degeneration on muscle MRI is also highly variable (21). The medial gastrocnemius severity is inconsistent with studies showing that the gluteus minimums and lumbar erector spinae are the most affected region (22). Besides, STIR sequences did not detect any myoedema in our cohort, while others reported that muscle edema was common (23), possibly because patient enrollment occurred at different stages of the disease.

A striking difference in MRI patterns between LGMD2B and MM was not observed. For example, patient 7 with MM showed increased signal in the proximal muscles (adductor magnus muscle and quadriceps femoris) and the distal gastrocnemius muscle, which demonstrates an initial impairment of both distal and proximal muscles regardless of the phenotype. However, recent literature shows that dysferlinopathy had a recognizable muscle MRI pattern, and disease duration can be described by muscle imaging using heatmaps and random forests (24). DACM patients had a unique MRI image that indicated selective involvement of anterior compartment muscles in the lower legs. The posterior compartment muscles were mostly unaffected even at late stages of the disease. However, since only one DACM patient was enrolled, a broader interpretation of the MRI results was limited, and more DACM patients require evaluation in the future.

Sarcoplasmic accumulation as one of four patterns of dysferlin staining in muscle biopsy specimens (normal, negative, faint, and abnormal cytoplasmic accumulation) should be paid more attention to. It must be noted here that because of sarcoplasmic labeling, sarcolemmal staining can result in a false-positive diagnosis. Therefore, additional protein studies (immunoblotting of dysferlin) should be performed to confirm the diagnosis or to guide further testing.

A reliable correlation of dysferlin expression in skeletal muscle and CD14^+^ peripheral blood monocytes (PBMs) has been established (25). However, whether total PBMC (without CD14^+^ cell isolation) could replace CD14^+^ PBMs is controversial. It has been demonstrated that insignificant differences exist in the dysferlin signal between isolated CD14^+^ PBMs and total PBMC (7). Moreover, relative dysferlin protein levels in total PBMC can be used to distinguish patients, carriers, and healthy individuals (7). CD14^+^ PBM isolation is an expensive step that has prevented smaller laboratories from using this less invasive protein assay for preliminary screening.

In this study, we successfully evaluated dysferlin expression in total PBMC. As expected, dysferlin levels were reduced in the total PBMC of patients, ranging from 26 to 39% of the control. However, the amount of dysferlin varied greatly among carriers (14–205%). Total PBMC dysferlin levels would overlap between levels in heterozygous carriers and patients, which contradicts another study (7) finding that relative dysferlin levels in total PBMC can be used to distinguish patients and carriers. Besides, our patients and carriers showed lower and higher dysferlin levels, respectively. Differences in antibodies (Abcam vs. NCL-Hamlet) and quantification methods used, in addition to individual heterogeneity, may explain this incongruity. Even the baseline of control individuals can vary among studies because of variations in dysferlin levels in healthy individuals (25). In addition, it is of note that two carriers (patient 2's father and mother) had lower than 20% dysferlin expression (14 and 19%, respectively), which may yield false results. Hence, it is cautious to use total PBMC as an alternative for evaluating dysferlin expression. We recommended that dysferlin levels in total PBMC may be useful in patients for whom muscle tissue is not available for study and to guide further testing in dysferlinopathies. Finally, owing to the small sample of total PBMC in patients, no correlation between the type and location of the mutation and the amount of protein in total PBMC was observed.

## Conclusion

In summary, this study presents novel *DYSF* mutations in a group of Chinese patients and expands the phenotypic spectrum of dysferlinopathies. One case with diagnostic ambiguity showed homozygous mutation in genomic DNA sequencing while heterozygous mutation in cDNA sequencing. Moreover, the study indicates total PBMC may serve as an alternative to muscle tissue when muscle tissues are unavailable, so as to guide further testing in dysferlinopathies. But it is still preliminary to use total PBMC as an alternative for evaluating dysferlin expression.

## Data Availability Statement

The data that support the findings of this study are available from the corresponding author upon reasonable request.

## Ethics Statement

The studies involving human participants were reviewed and approved by Guangzhou first people's hospital ethics committee. The patients/participants provided their written informed consent to participate in this study.

## Author Contributions

ZL and HZ contributed to study design, the drafting and revision of the manuscript, data acquisition, and analysis. YL contributed to the methodology and contributed to genetic information analysis. QC contributed to patient's recruitment, data acquisition, and manuscript revision. XC contributed to patient's recruitment and data acquisition. All authors read and approved the manuscript.

## Conflict of Interest

The authors declare that the research was conducted in the absence of any commercial or financial relationships that could be construed as a potential conflict of interest.
